# Investigating metropolitan change through mathematical morphology and a dynamic factor analysis of structural and functional land-use indicators

**DOI:** 10.1038/s41598-023-27686-1

**Published:** 2023-01-13

**Authors:** Samaneh Sadat Nickayin, Gianluca Egidi, Pavel Cudlin, Luca Salvati

**Affiliations:** 1 Planning and Design Faculty, Agricultural University of Iceland, 311 Hvanneyri, Borgarbyggð, Iceland; 2 Department of Agricultural and Forest Sciences, University of Tuscia, Via S. Camillo de Lellis, 01100 Viterbo, Italy; 3 Global Change Research Institute, Lipová 1789/9, 370 05 Ceske Budejovice, Czech Republic; 4 Department of Methods and Models for Economics, Territory and Finance (MEMOTEF), Faculty of Economics, Sapienza University of Rome, Via Castro Laurenziano 9, I-00161 Rome, Italy

**Keywords:** Ecology, Ecology, Environmental sciences, Environmental social sciences

## Abstract

We presented an operational rationale grounded on complex system thinking to quantify structural and functional landscape transformations along three stages representative of post-war metropolitan development in Rome, Italy (urbanisation with population/settlement densification, 1949–1974; suburbanisation with medium-density settlement expansion, 1974–1999; counter-urbanisation with settlement sprawl, 1999–2016). A mathematical morphology approach assessing the geometric form of land patches and a multi-way factor analysis (MFA) of landscape metrics were used to investigate the joint evolution of urban form and land-use functions over time. The empirical results of the MFA delineated the multivariate relationship between nine land-use classes (with distinctive socioeconomic functions) and seven morphological types (reflecting different landscape structures) according to four observation times (1949, 1974, 1999, 2016). Taken as an intrinsic attribute of complex landscape systems experiencing intense transformations, an estimation of the ‘rapidity-of-change’ in the form-functions relationship at a given development stage was derived from MFA outcomes separately for urbanisation, suburbanisation, and counter-urbanisation. A simplified form-functions relationship, reflecting the spatial polarisation in compact settlements and rural (low-density) landscapes, was observed with compact urbanisation. By stimulating urban sprawl into fringe farmland, suburbanisation resulted in patchy and heterogeneous rural landscapes. Counter-urbanization was associated with the fragmentation of built-up settlements leading to a chaotic mosaic of land structures that mixes urban and rural traits. Rapidity-of-change in form-function relationships was greater during suburbanisation than urbanisation and counter-urbanisation. It reflects the intrinsic pressure of economic growth in contemporary cities.

## Introduction

Urban growth in advanced countries was demonstrated to cause subtle landscape transformations^1–4^. Patch fragmentation, spatial polarisation in urban and non-urban land, simplification and homologation of natural landscapes are transformative processes characteristic of suburban and rural districts experiencing economic growth, population increase, and settlement expansion^5–8^. The resulting landscape matrix became particularly complex and spatially entropic, with a massive increase in the fractal dimension of individual patches as one of the most evident attributes of change^9–12^. Although the influence of compact urbanisation on landscape structure and composition is relatively well known^13–17^, less explored is the specific impact of sprawl on dense cities with socioeconomic functions expanding into suburban locations^18–21^.

In such contexts, the design of empirical models and operational frameworks to quantify and understand the long-term evolution of landscape systems in metropolitan regions is a challenging task^22–25^. Every approach should ensure theoretical parsimony and consistency with the state of knowledge^26–28^. The present study assumes the metropolitan landscape as a Complex Adaptive System (CAS) that reflects a continuous interplay between biophysical conditions and rapidly evolving human contexts at the local scale^29^. In other words, metropolitan landscapes can be envisaged as “open systems shaped by nonlinear dynamics involving agents capable of anticipation and emerging types of spatial units”^30^.

With this perspective in mind, CAS thinking is considered a valid (interpretative and operational) approach to evolving landscapes where multiple agents of change interact via complex (multivariate) relationships that determine nonlinear, mostly unpredictable feedback among the composing dimensions of a given system^31–33^. The interaction between economic agents produces new spaces at two observation levels^34–36^, affecting (i) the morphological structure that derives from pristine landscapes, territorial/planning constraints, and ecological conditions, and (ii) the spatial organisation of population and economic activities. Such results in a complex land-use matrix that reflects citizens’ decisions, adaptive strategies, competitive relations, and feedback interactions between systems’ dimensions^3,37,38^.

The selection of the intrinsic properties describing the evolution of metropolitan regions is another crucial point in a ‘complex system thinking’^39^. In this perspective, the relationship between (urban) form and (socioeconomic) functions was assumed as a key property of a given landscape^40^. However, despite being investigated extensively in recent times, there is no consensus on the most significant conceptual dimensions and measurement approaches (e.g. indicators) in land-use science^41–43^. An operational solution is to assume landscape dynamics as determined by limiting/controlling factors (‘slow’ variables) while the CAS moves around the regime (i.e. changes state) depending on the values of ‘fast’ drivers^44–46^. Classified as a CAS low-level property, ‘rapidity-of-change’ (i.e. “the capacity to meet priorities and to achieve goals promptly to contain losses and thwart future disruption”^38^) was operationally defined as the result of the intimate interplay between ‘fast’ and ‘slow’ variables characteristic of a given landscape system^30,47,48^.

The present study adopts a CAS thinking to investigate long-term changes in structures and functions taken as the main components of landscape dynamics in metropolitan regions with a multi-way factor analysis of indicators derived from a Morphological Spatial Pattern Analysis, hereafter MSPA^49,50^. Landscape structure was investigated through quantitative indicators^51–53^ derived from applying a MSPA to high-resolution digital maps^54–56^. Landscape functions were analysed, distinguishing classes with specific land values (e.g. urban settlements) or representative of distinctive (agricultural/forestry) productions with high (e.g. vineyards) or low (e.g. pastures) economic potential^48,57,58^. This classification was also intended to discriminate land use with different exploitation levels^1,32,59^, reflecting a high (e.g. arable land) or low (e.g. olive groves) intensity of human use^60–62^ as well as environmental impact, e.g. mechanisation, irrigation, pest control^12^.

Translating the logical framework into an operable approach^31^, landscape transformations were interpreted considering together changes in (spatial) structure and (land-use) functions over a sufficiently long period (1949–2016) in a case study (Rome, Central Italy) representative of compact cities in Mediterranean Europe progressively going toward sprawl^63–65^. More specifically, the operational framework was aimed at (i) identifying the most significant (morphological and structural) characteristics of Rome’s landscape and evaluating their linkage with urban development, (ii) inferring the form-function relationship at different growth stages, and (iii) delineating territorial dynamics common to other cities in advanced economies under similar territorial contexts^66–68^. The novelty of this approach lies in the holistic assessment of landscape change on a metropolitan scale with respect to the intrinsic timing of urban expansion^20,36,69^. Among economic, historical and geographical theories of urban growth^70^, the City Life Cycle (CLC) was considered a suitable framework for exploring the relationship between urban/exurban development, economic structure, and socio-demographic aspects at both local and regional observation scales^71^. By identifying and profiling sequential expansion stages, CLC defines a ‘metropolitan cycle’ constituted of distinctive ‘waves’ (i.e. homogeneous time intervals during which a developmental phase emerges and declines), and ‘transitions’ (intended as the (shorter) time interval between two waves). Empirical exercises derived from a comprehensive analysis of changes over time in the total population of inner cities and surrounding areas define some standard stages (e.g. urbanisation, suburbanisation, counter-urbanisation, and re-urbanisation) characteristic of CLCs in advanced economies, and especially in Europe^20^.

Based on these premises, our study was organised in four steps. Following CLC theory, we initially made use of a large set of landscape indicators to confirm the timing of post-war development (urbanisation, suburbanisation, counter-urbanisation) that was delineated *ex-ante* in the study area (metropolitan Rome) based on comparative scrutiny of recent literature. Considering separately the three developmental stages mentioned above, the subsequent analysis decomposed landscape changes in two operational dimensions—namely morphological (i.e. land structure) and functional (i.e. land-use), using a set of quantitative metrics derived from MSPA. A Multi-way Factor Analysis (MFA) was consequently run on the whole set of landscape indicators to depict the joint evolution of structural and land-use dimensions assumed to reflect dynamic form-functions relationships at each developmental stage. Highlighting the potential for landscape transformations inherent in the three developmental stages, a standardised metric was finally derived from MFA results to quantify long-term ‘rapidity-of-change’ understood as an intrinsic property of CASs.

## Methodology

### Study area

**Figure 1:**
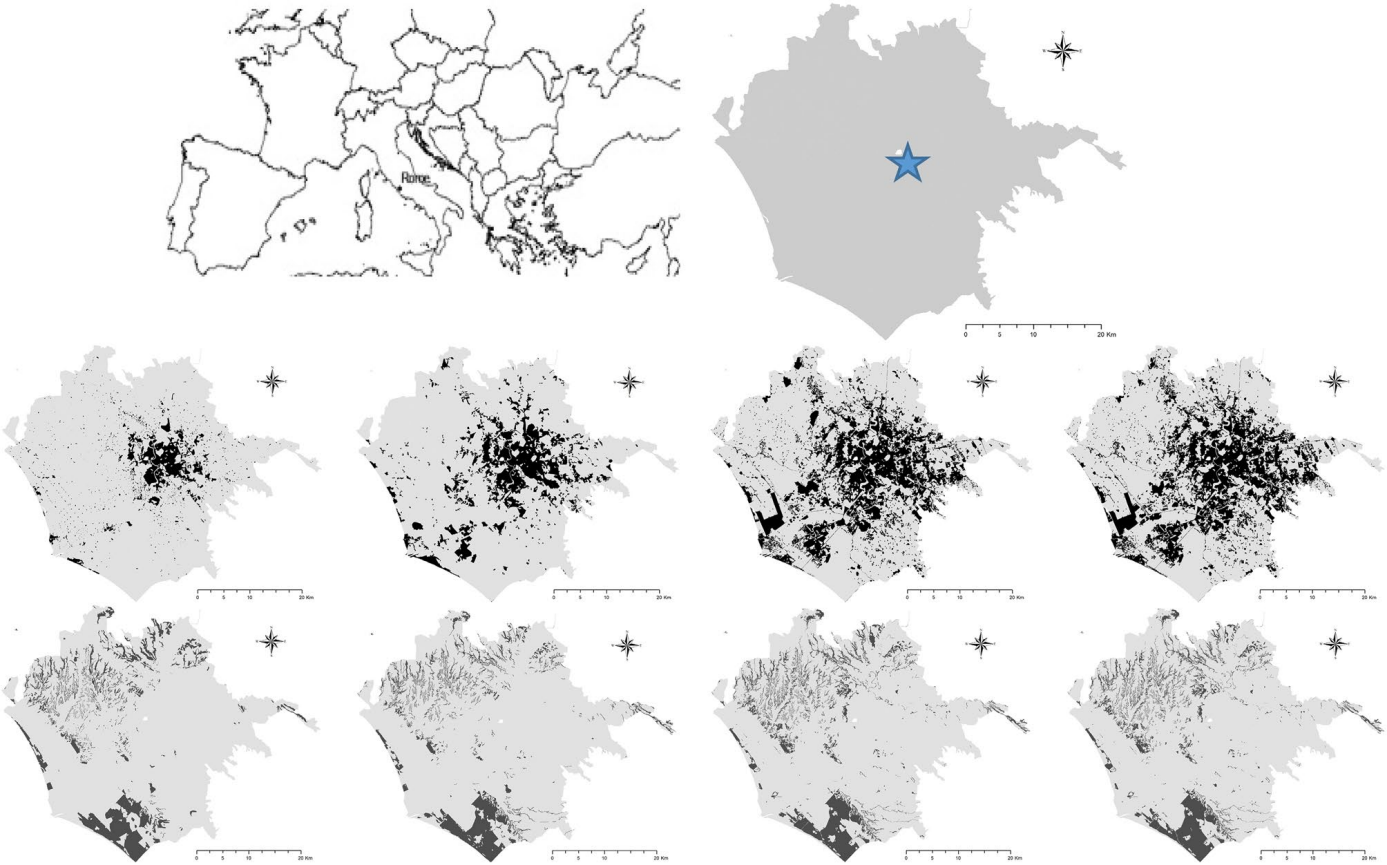
Landscape transformations in Rome (1949–2016); upper panel: the geographical position of the study area in Europe (left) and the extent of the study area, with the star indicating downtown Rome (right). Middle panels, from left to right: the spatial distribution of urban areas in 1949, 1974, 1999 and 2016; lower panel, from left to right: the spatial distribution of woodlands in 1949, 1974, 1999 and 2016 (the maps were created with the software ArcGis 9.3 from the shapefiles described in the methodological chapter).

We investigated landscape dynamics over nearly 70 years (1949–2016) in a metropolitan region of Central Italy encompassing the municipalities of Rome and Fiumicino (Latium region) and covering a total surface area of 1497 km^2^^13^ with a primarily flat topography (90% lowlands, 10% uplands). The flat area, placed over the alluvial plain of the Tiber River^72^, corresponded in large part with the rural district better known as ‘Agro Romano’^73^, being a room of compact-dense settlements in its central part (inner Rome). Industrial areas are primarily located in the eastern part of the ‘Agro Romano’^36^; natural landscapes concentrate in the western part of the area^74^. Although urban settlements occupy a large part of the study area (Fig. 1), forests, pastures, and cultivated land are still common^63^. According to earlier studies^12^, the expansion of compact settlements was primarily observed between the early-1950s and the mid-1970s^70^; population increase was more evident in central districts than in the suburbs—corresponding with a classical ‘urbanisation’ stage of the CLC^64^. The dispersed urban expansion was observed since the mid-1970s as being characteristic of the ‘suburbanisation’ stage^75^. The population density gap in urban and rural areas reduced over time, and the ratio of suburban-to-urban population increased from 25% (1981) to 34% (2001). Since the late 1990s, the population has decreased in central districts while rising in suburban locations^17^. Consequently, overall population density increased from 1104 inhabitants/km^2^ (1951) to 1935 inhabitants/km^2^ (2021).

#### Identifying individual stages of Rome’s life cycle

Three-time intervals were identified to investigate the impact of sequential developmental stages on urban form and socioeconomic functions^36^. Following the CLC theory^20^, empirical evidence from earlier studies^17,63,70,76^ allowed the identification of three stages that reflect differentiated demographic and settlement dynamics in Rome^77^. With ‘urbanisation’ (1949–1974), a massive population increase was recorded together with intense (compact) building development^13^. With ‘suburbanisation’ (1974–1999), population growth slowed down, as opposed to the rate of land conversion into residential settlements^63^, as built-up areas mainly expanded into rural areas in a primarily discontinuous way^72^. A slight demographic recovery and a moderate slowdown in the rate of land consumption were finally observed during the ‘counter-urbanisation’ (1999–2016) stage^70^.

A selection of quantitative indicators (Table 1) aimed to delineate the characteristic territorial profile of the three developmental stages described above, since they are hypothesised to represent distinctive trends in landscape composition and structure over time. These trends are, in turn, assumed to influence (i) the spatial arrangement of settlements and (ii) the relationship with population density, among others^23^. Settlements extended 6.6% of the study area in 1949 and expanded to 15.9% in 1974, covering 26% and 28% of the study area, respectively, in 1999 and 2016. The total population increased from 1.6 million inhabitants in 1951 to 2.9 million inhabitants in 2021^17^.

**Table 1:** Per cent share of ‘core’ area in total landscape by land-use class in metropolitan Rome (panel a) and selected territorial variables, 1949–2016 (panel b).

(a) Land-use	Absolute (or per cent) values				Annual change over time (%)		
	1949	1974	1999	2016	1949–1974	1974–1999	1999–2016
Share (%) of core area in total landscape							
Built-up area	34.9	42.2	36.9	35.5	0.84	− 0.51	− 0.21
Urban green	30.5	27.2	19.5	19.2	− 0.42	− 1.13	− 0.10
Arable land	61.6	50.4	37.4	35.4	− 0.73	− 1.03	− 0.30
Crop mosaic	17.1	16.3	4.2	5.4	− 0.20	− 2.98	1.68
Vineyards	42.9	32.5	8.7	8.5	− 0.97	− 2.93	− 0.17
Olive groves	14.0	7.6	1.7	3.1	− 1.83	− 3.12	5.15
Woodland	36.6	34.6	29.2	29.2	− 0.22	− 0.63	0.01
Pastures	25.2	23.5	6.9	6.5	− 0.28	− 2.82	− 0.34
Wetlands	1.6	2.4	2.2	2.1	2.10	− 0.35	− 0.13
(b) Territorial attributes							
Population density (inh/km^2^)	1103	1848	1875	1934	2.3	0.1	0.2
Share of built-up areas in total landscape (%)	6.6	14.8	25.9	27.6	5.1	3.0	0.7
Per-capita built-up area (%)	0.59	0.80	1.39	1.54	1.4	2.9	1.2

The population overgrew during ‘compact urbanisation’ and less markedly during both ‘suburbanisation’ and ‘counter-urbanisation’^73^. Economic informality and spontaneous building expansion led to dense (or semi-dense) settlements between 1949 and 1974^36^. Rising from 59 m^2^ (1949) to 153 m^2^ (2016), the amount of per-capita built-up area accelerated with suburbanisation, together with the expansion of traditional tertiary sectors (construction, commerce, public services) and the increasing importance of residential sub-centres^4^. With counter-urbanisation, the role of the public sector in shaping the form and functions of the inner city declined substantially^17^, giving room to a parallel expansion of value-added creative sectors (including tourism) with high-qualification jobs^64^. Sprawl intensity reduced slightly compared with suburbanisation, likely because of the increased effectiveness of spatial planning^78^ and the indirect impact of the great recession on land and housing prices—declining mainly in central areas^75^.

### Data sources

Based on a minimum mapping unit of 1 hectare, landscape indicators were extracted from comparable maps and geo-spatial databases officially produced by regional and national authorities/institutions in Italy and using a nomenclature system inspired by Corine Land Cover (CORINE) classification^79^. The information sources adopted in this study include a topographic map realised by the Italian Istituto Geografico Militare (IGM) referring to 1949 and the ‘Agricultural and forest map of Rome province’ released by the Cartographical Service of Rome’s province and dated back to 1974^23^. Two versions of the 'land-use map of Latium region' (produced by the Cartographic Service of the Regional Authority of Latium through interpretation of digital ortho-photographs dated 1999 and 2016) were also considered.

The use of these data sources in long-term landscape analysis was extensively discussed in earlier studies dealing with Rome's environmental and socioeconomic transformations^63,64,74,76^. We adopted national, regional and local official maps instead of global- and continental-scale maps with a broader nomenclature system but a shorter temporal coverage and less spatial detail^19^. Using public data from regional/local sources further represents an indirect assessment of the stability, reliability, and flexibility of the model, as such information sources in advanced economies are by far more frequently available than sources with global (or continental) coverage^4^.

### Assessing landscape dynamics through the identification of structures and functions

A diachronic landscape analysis based on quantitative metrics was carried out in this study to elucidate the long-term path of settlement expansion in Rome, the consequent trajectories of change in the surrounding (non-urban) landscapes, and the related socioeconomic context characteristic of each developmental stage^80–82^. Selection of elementary variables, identification of relevant analysis dimensions, and construction of composite indicators adequate to describe changes over time in landscape morphology and functions have been subsequently set up following criteria of comprehensiveness, reliability, and calculation easiness^22,83,84^. More specifically, two dimensions of a given landscape system have been explored in this study: ' functions’ and ‘structure’^85^. Nine land-use classes were adopted to comprehensively describe relevant (socioeconomic) functions in the landscape^23^. Seven structural metrics computed for each land-use class have been selected to provide a complete description of landscape morphology^86^. Considering together configuration and compositional aspects of a given landscape matrix, these metrics were representative of multiple dimensions such as fragmentation, patch shape, fractal dimension, and mosaic complexity^87^.

#### Landscape functions

Nine basic classes were considered in this study as representative of the most frequent land use in Rome^63^: (i) built-up settlements, (ii) urban green, (iii) arable land, (iv) crop mosaic, (v) vineyards, (vi) olive groves, (vii) woodland, (viii) pastures, and (ix) wetlands. These classes were also assumed to reflect significant economic functions (e.g. urban, agriculture, forestry) in the area^12^, being able to discriminate individual land-use with different exploitation intensity (e.g. arable land *vs* pastures) or economic value (e.g. vineyards *vs* woodland), according with Salvati et al.^64^. These classes were extracted from the digital maps described above (“Data sources” section) through the implementation of simple aggregation/disaggregation (e.g. union, intersection) rules available in computational tools for ArcGIS (release 10) software (ESRI Inc., Redwoods, USA) and the ‘Patch Analyst’ extension for ArcGIS 9.3^4^. The final nomenclature system was derived from earlier studies^23^ and assumed to be comprehensible to stakeholders, planners, and practitioners not confident with spatial analysis, geographic information systems, and remote sensing tools^57,60^.

#### Landscape structure

The landscape matrix was segmented into structural types separately for each land-use function using multivariate mathematical morphology^33^, a quantitative framework that analyses the shape and form of a given landscape object^88^. We specifically adopted a Morphological Spatial Pattern Analysis (MSPA) implementing image processing routines that identify hubs, links (i.e. corridors), and other features relevant to a structural assessment of landscapes^89^. MSPA was selected for its flexibility and scientific soundness tested in earlier works^49,50,54–56^. While rigorous and documented widely, the approach is simplified enough to be applied within non-technical contexts (practitioners, planners, ecologists, and general stakeholders).

Additionally, MSPA was adopted in this study because it refers specifically to a broader, operational concept of landscape structure associated with the entire (regional) scene and is not limited to the urban (settlement) matrix^90^. In other words, we are particularly interested in investigating (non-urban) landscape changes using urban transformations as a sort of ‘timing’ (background) variable, i.e. outlining landscape dynamics along three different stages of post-war urban development. Since MSPA assures a global analysis of landscape structure using simplified, flexible and readable metrics that can be analysed further through exploratory multivariate statistics (as we will explain later on in “Tracing the evolution of a complex landscape system” section), this approach is appropriate to the aims and scope of our study. However, other frameworks can be considered when analysing urban morphology and specific landscape structures, both model-based and indicator-based^22,32,51,85^, both theory-driven and exquisitely empirical^11,15,35,80^.

MSPA identified seven morphological typologies (cores, perforations, islets, bridges, loops, branches, edges) for each land-use class^55^. ‘Cores’ are defined as the inner part beyond a certain distance to the boundary^49^. ‘Islets’ are those parcels that are too small (and isolated) to form a core area defined as above^50^. ‘Edges’ and ‘perforations’ surround each core area^91^; more specifically, ‘perforations’ are identified as the transition zone between ‘cores’ and a different land-use class^54^; ‘edges’ represent in turn a transition between ‘core’ and ‘non-core’ patches within the same land-use class^49^. Finally, loops, bridges, and branches are small and mostly convoluted patches connecting core areas^39^. More specifically, ‘loops’ are corridors connecting the same core patch^78^, ‘bridges’ connect at least two different cores^89^, and ‘branches’ connect a core area with a non-core area within the same land-use class^90^.

Using Guidos software, landscape classification based on MSPA was realised by computation on the spatial distribution of the nine land-use classes (shapefiles) appropriately rasterised using the ‘spatial analyst’ tool available in the ArcGIS package^37^. The surface area of the seven MSPA types (see above) was calculated separately for each year (1949, 1974, 1999, 2016) and land-use class^92^. MSPA processes identify core areas based on connectivity rules defining neighbours and the value used to define edge width^55^. Consequently, connectivity was set for a given pixel node to its adjacent pixels by considering eight neighbours (i.e. a pixel border and a pixel corner in common), allowing identification of the remaining landscape categories^54^.

### Tracing the evolution of a complex landscape system

Covering a wide range of spatial patterns, the joint analysis of land-use classes (functions) and MSPA types (morphology) allows a comprehensive investigation of the form-function relationship^93^ at the base of landscape transformations in Rome. The proposed framework was articulated in three steps. First, an exploratory, dynamic analysis (the so-called Multi-way Factor Analysis, MFA) of two landscape dimensions (structure and functions) and their latent (multivariate) relationship provided an indirect assessment of low-level properties characteristic of a Complex Adaptive System (e.g. connectedness, redundancy). Second, structural and functional attributes of the landscape were discriminated into ‘fast’ and ‘slow’ variables, separately for urbanisation, suburbanisation, and counter-urbanisation stages. Third, a metric of a rapidity-of-change characteristic of the whole landscape system was estimated from computation on specific analysis’ outputs. Based on the joint analysis of changes in all the elements composing the landscape system^94^, MFA allows evaluating if the position of each unit (i.e. structural class) or case (i.e. functional class) is stable or variable over time by projecting them into the same factorial plane^95^. This procedure allows identification of the studied system's ‘fast’ and ‘slow’ attributes, providing a global estimation of rapidity-of-change^30^.

#### A dynamic analysis of landscape transformations with structural and functional indicators

By decomposing structural from functional changes, a MFA of seven structural (morphology) classes (columns) and nine functional (land-use) classes (rows) was run considering four years (1949, 1974, 1999, 2016). The average patch size (ha) by structure and function (for instance, the mean size of ‘core’ patches of urban settlements or the mean size of ‘loop’ patches of olive groves) was the specific value in each cell of the input matrix that was subjected to MFA, after data standardisation^59^. Dynamic multi-dimensional analysis’ techniques capture complex structures in higher-order datasets—where data have more than two dimensions^30^. In our case, MFA decomposed landscape changes into three dimensions (structure, functions, and developmental stage). By associating different variables with similar spatio-temporal patterns on a few significant axes, this analysis provides an indirect measure of redundancy^47^, or the extent to which the system’s elements (i.e. indicators) have substitutes to ensure functioning in the event of a transition or a shock^45^.

Belonging to the broad family of factorial techniques, MFA is a generalisation of exploratory multivariate statistics such as the Principal Component Analysis of variables collected on the same set of observations^96^. MFA allows a comparative investigation of the relationship between the different data sets over time, identifying a standard data structure called ‘compromise’—which is then analysed via spectral decomposition of the input matrix, revealing common structures between the observations^95^. Each data set was projected into the ‘compromise’ space to analyse commonalities and discrepancies^30^. The ‘compromise’ weights^97^ were chosen maximising the representativeness of all four data sets (i.e. 1949, 1974, 1999, 2016). Significant factors with eigenvalues > 1 were selected for analysis. This criterion considers factors that extract a satisfactory proportion of variance from the input data matrix ^23^.

#### Estimating the overall ‘rapidity-of-change’ of a given landscape system

A comprehensive framework identifying ‘fast’ and ‘slow’ dimensions underlying structural and functional transformations^98^ and estimating the (overall) ‘rapidity-of-change’ in a complex landscape system was proposed here^99^. A multivariate measure of rapidity-of-change (R’) for each structural and functional class was calculated as the Euclidean, the n-dimensional distance between loadings (or scores) observed at times t_x+1_ and t_x_ (e.g. 1974 vs. 1949, namely ‘urbanisation’):

$$R^{\prime} = \surd {{\left( {\left( {{\text{x}}_{1,1} - x_{1,0} } \right)^{2} + \left( {{\text{x}}_{2,1} - {\text{x}}_{2,0} } \right)^{2} + \left( {{\text{x}}_{ \ldots ,1} - {\text{x}}_{ \ldots ,0} } \right)^{2} + \left( {{\text{x}}_{n,1} - {\text{x}}_{n,0} } \right)^{2} } \right)} \mathord{\left/ {\vphantom {{\left( {\left( {{\text{x}}_{1,1} - x_{1,0} } \right)^{2} + \left( {{\text{x}}_{2,1} - {\text{x}}_{2,0} } \right)^{2} + \left( {{\text{x}}_{ \ldots ,1} - {\text{x}}_{ \ldots ,0} } \right)^{2} + \left( {{\text{x}}_{n,1} - {\text{x}}_{n,0} } \right)^{2} } \right)} t}} \right. \kern-0pt} t}$$

where x_a,b_ is the loading on factor a at time b, n is the number of factors with eigenvalues > 1, and *t* is the length of each time interval, expressed as the total number of years. Fast and slow variables and rapidity-of-change were thus investigated separately for two-time horizons^30^: (i) a short-term time window, i.e. considering separately each growth stage, namely urbanisation, suburbanisation, and counter-urbanisation) and (ii) a long-term time window, i.e. considering the metropolitan cycle between 1949 and 2016. Fast and slow attributes were defined as having an above-median or below-median rapidity of change calculated for each dimension (structure and functions) separately^77^. These metrics ultimately aimed at estimating the contribution of these two dimensions to the overall system’s evolution^3,44,100^.

## Results

### Landscape dynamics and developmental stages in Rome, 1949–2016

A descriptive analysis of landscape transformations in metropolitan Rome (1949–2016) was reported in Tables 1, 2 and 3. We initially considered the per cent share of ‘core’ areas in total class area (and the relative (annual) rate of change) as a summary indicator of urban growth and landscape modifications (Table 1) calculated for each land-use class and developmental stage (urbanisation: 1949–1974; suburbanisation: 1974–1999; counter-urbanisation: 1999–2016). The share of built-up settlements classified as ‘core’ patches in the total class area experienced a marked increase with urbanisation (+ 0.8% per year) and reached the highest value in 1974 (42 ha). These dynamics reflect settlement densification on a local scale and spatial polarisation in urban and rural functions on a regional scale. With suburbanisation, the share of ‘core’ built-up patches in the total class area decreased rapidly (− 0.5% per year). The counter-urbanisation stage was associated with a further decrease in the share of ‘core’ built-up patches (− 0.2% per year), bringing the 2016 (average) value back to the 1949 level. Altogether, the long-term dynamics of built-up patches classified as ‘core areas’ reflect a process of population dispersion resulting from the expansion of spatially discontinuous and low-density settlements.

**Table 2:** Mean patch size (ha) by year and structural (rows)/functional (columns) class in metropolitan Rome.

Class	Built-up area	Urban green	Arable land	Crop mosaic	Vineyards	Olive groves	Woodland	Pastures	Wetlands
1949									
Core	20.3	23.3	73.2	6.7	33.1	6.4	21.8	8.6	15.0
Islet	0.9	6.8	1.5	2.2	1.5	4.6	2.0	3.3	7.1
Perforation	6.0	0.0	3.4	0.0	1.3	0.0	10.0	6.7	0.0
Edge	9.0	26.6	3.6	8.6	12.2	13.9	7.8	7.6	15.0
Loop	5.3	15.0	4.8	7.5	7.5	15.0	8.2	5.0	0.0
Bridge	6.7	10.4	24.0	7.5	6.2	0.0	8.4	9.8	0.0
Branch	1.1	2.9	0.7	1.5	1.2	2.9	1.7	1.5	2.5
1974									
Core	23.3	20.7	35.7	4.3	14.4	2.9	30.7	7.2	5.0
Islet	3.2	7.0	2.4	3.1	3.5	3.4	3.3	3.2	3.1
Perforation	8.2	0.0	5.5	0.0	0.0	0.0	4.5	0.0	0.0
Edge	8.5	17.3	4.5	7.4	8.6	8.4	5.9	6.4	15.0
Loop	5.2	25.0	4.6	6.0	5.4	15.0	9.0	6.5	0.0
Bridge	7.9	19.0	14.8	5.4	8.9	3.7	13.7	8.2	0.0
Branch	1.1	2.0	0.7	1.1	1.2	1.4	1.1	1.2	2.5
1999									
Core	17.8	10.2	15.9	1.6	2.6	1.4	24.5	2.4	7.5
Islet	1.4	2.7	1.8	2.1	2.0	1.8	1.8	2.3	3.7
Perforation	5.5	0.0	4.4	0.0	0.0	0.0	10.7	0.0	0.0
Edge	4.7	8.9	2.7	5.4	2.7	3.7	4.2	4.0	15.0
Loop	5.9	5.4	5.3	10.0	5.0	7.5	12.8	7.6	0.0
Bridge	9.8	10.6	24.3	10.0	24.3	7.5	24.3	10.1	0.0
Branch	0.9	1.6	0.7	1.3	0.9	1.5	1.1	1.6	2.1
2016									
Core	16.3	10.1	14.9	1.9	2.3	2.1	24.0	2.3	7.5
Islet	1.5	2.8	1.8	2.1	1.8	1.5	1.8	2.0	3.8
Perforation	5.0	0.0	4.3	0.0	0.0	0.0	7.5	0.0	0.0
Edge	4.1	9.6	2.8	5.7	2.8	3.0	4.3	3.5	15.0
Loop	5.5	6.9	5.5	6.4	5.0	7.5	12.1	8.5	0.0
Bridge	11.1	10.0	23.9	10.7	26.2	15.0	22.1	10.1	0.0
Branch	0.9	1.6	0.8	1.2	1.0	1.5	1.2	1.4	2.0

**Table 3:** Per cent annual change over time (%) in the mean patch size by developmental stage and structural (rows)/functional (columns) class in metropolitan Rome.

Class	Built-up area	Urban green	Arable land	Crop mosaic	Vineyards	Olive groves	Woodland	Pastures	Wetlands
Urbanization									
Core	0.12	− 0.11	− 1.50	− 0.10	− 0.74	− 0.14	0.36	− 0.06	− 0.40
Islet	0.09	0.01	0.03	0.04	0.08	− 0.05	0.05	0.00	− 0.16
Perforation	0.09	0.00	0.08	0.00	− 0.05	0.00	− 0.22	− 0.27	0.00
Edge	− 0.02	− 0.37	0.04	− 0.05	− 0.14	− 0.22	− 0.08	− 0.05	0.00
Loop	0.00	0.40	0.00	− 0.06	− 0.08	0.00	0.03	0.06	0.00
Bridge	0.05	0.34	− 0.37	− 0.08	0.10	0.15	0.21	− 0.06	0.00
Branch	0.00	− 0.04	0.00	− 0.02	0.00	− 0.06	− 0.02	− 0.01	0.00
Suburbanization									
Core	− 0.22	− 0.42	− 0.79	− 0.11	− 0.47	− 0.06	− 0.24	− 0.19	0.10
Islet	− 0.07	− 0.17	− 0.02	− 0.04	− 0.06	− 0.07	− 0.06	− 0.04	0.02
Perforation	− 0.11	0.00	− 0.04	0.00	0.00	0.00	0.25	0.00	0.00
Edge	− 0.15	− 0.33	− 0.07	− 0.08	− 0.24	− 0.19	− 0.07	− 0.10	0.00
Loop	0.03	− 0.78	0.03	0.16	− 0.02	− 0.30	0.15	0.04	0.00
Bridge	0.08	− 0.34	0.38	0.18	0.62	0.15	0.42	0.08	0.00
Branch	− 0.01	− 0.02	0.00	0.01	− 0.01	0.01	0.00	0.01	− 0.02
Counter-urbanziation									
Core	− 0.09	0.00	− 0.06	0.02	− 0.02	0.05	− 0.03	− 0.01	0.00
Islet	0.00	0.01	0.00	0.00	− 0.01	− 0.01	0.00	− 0.02	0.00
Perforation	− 0.03	0.00	0.00	0.00	0.00	0.00	− 0.19	0.00	0.00
Edge	− 0.03	0.04	0.01	0.02	0.00	− 0.04	0.00	− 0.03	0.00
Loop	− 0.03	0.09	0.01	− 0.21	0.00	0.00	− 0.04	0.05	0.00
Bridge	0.08	− 0.03	− 0.02	0.04	0.11	0.44	− 0.13	0.00	0.00
Branch	0.00	0.00	0.00	0.00	0.01	0.00	0.00	− 0.01	− 0.01

Changes over time in the average patch size by developmental stage and land-use class were subsequently elaborated and provided further insight into the descriptive analysis of long-term landscape changes in Rome. The average patch size of built-up areas increased from 20 ha (1949) to 23 ha (1974) and decreased afterwards (18 ha: 1999; 16 ha: 2016). Non-urban land-use classes showed three distinctive trends, possibly depending on urban dynamics. The average patch size of green (urban) areas, arable land, vineyards, and pastures decreased continuously over time. The average patch size of crop mosaics, olive groves, and woodlands experienced a net decrease during both urbanisation and suburbanisation, and a moderate recovery with counter-urbanisation. Wetlands and water bodies showed a moderate increase with urbanisation and a slight decrease during suburbanisation and counter-urbanisation.

A specific analysis of the individual dynamics over time demonstrates how land-use classes respond to multiple drivers and contextual factors of change, being vastly different over time (developmental stage) and space (morphological category). For instance, the average size of green urban areas has undergone a continuous contraction over time, declining from 30.5 ha (1949) to 19.2 ha (2016). The most significant decrease was observed with suburbanisation and testified to the progressive fragmentation of built-up settlements and ancillary surfaces (e.g. gardens and parks) in Rome. A similar dynamic was observed for arable lands, which experienced a considerable decrease in the average patch size from 62 ha (1949) to 35 ha (2016). As in the case of green urban areas, the most intense decrease was observed with suburbanisation. Since arable land has represented the dominant crop in the study area for centuries, these results may outline the progressive fragmentation of rural landscapes after World War II.

Vineyards—another dominant use of agricultural land in contemporary Rome—underwent a similar contraction during the study period. More specifically, the average size of vineyards decreased from 43 ha (1949) to 8.5 ha (2016). The most rapid decrease was observed, once again, with suburbanisation (− 2.9%)—being less intense during urbanisation (-1%), and relatively modest during counter-urbanisation (-0.2%). The average size of pastures also reduced systematically from 25.2 ha in 1949 to 6.5 ha in 2016. The largest decrease was observed during suburbanisation (− 2.9%).

From an average patch size of 17 ha in 1949 to only 4 ha in 1999, crop mosaics underwent a progressive fragmentation between 1949 and 1999—a process more intense than for other (non-urban) land-use classes and particularly evident during suburbanisation (− 3%). In contrast, the average patch size of crop mosaics increased weakly with counter-urbanisation (+ 1.7%), and reflects the emerging complexity of peri-urban landscapes. The rapid decrease in the average size of olive grove patches recorded during both urbanisation (− 1.8%) and suburbanisation (− 3.1%), contrasts with the sharp recovery observed in the counter-urbanisation stage (+ 5.2%). Woodlands experienced a moderate decline in the average patch size over the first two developmental stages and substantial stability in the third stage.

On the basis of descriptive statistics of the average path size by morphological class (structure) and land-use (functions), Table 2 (reporting the absolute values by observation year) and 3 (reporting the per cent annual rate of change) illustrate landscape dynamics in metropolitan Rome, decomposing structural and functional changes separately for the three developmental stages. All the morphological types referring to built-up areas showed a net increase during urbanisation, except edges. On the contrary, during suburbanisation, all morphological types (referring to both urban and non-urban land use) showed a (more or less evident) reduction in size (i.e. surface area), with the only exception of loops and bridges. With counter-urbanisation, morphological classes experienced a slight reduction (or substantial stability) in the average patch size, except for bridges.

### A dynamic analysis of structural and functional indicators of landscape change

MFA extracted three principal axes for 80.2% of the total variance (Table 4). The first axis explained 46.6% of the overall variance, while the second and third axes captured 18.8% and 14.8% of the total variance, respectively. Factorial axes from the fourth on have secured a modest contribution to the total system’s variance and have not been considered further in this analysis. MFA projected variables and cases on the same plane separately for the four observation years (1949, 1974, 1999, 2016). Variables and cases corresponded with the seven morphological types and the nine land-use classes characterising the overall landscape matrix. The MFA biplot allows the delineation of landscape dynamics over time, considering variations in structure and functions.

**Table 4:** Results of a dynamic Multi-way Factor Analysis (MFA) resuming changes over time in structural and functional (landscape) indicators over the first three Axes (A1–A3) of the factorial plane in metropolitan Rome, by year.

Variable	1949			1974			1999			2016		
	A1	A2	A3	A1	A2	A3	A1	A2	A3	A1	A2	A3
Structure												
Core	0.45	0.38	0.32	0.64	0.71	0.24	0.55	0.66	0.41	0.52	0.68	0.39
Islet	− 0.89	0.30	0.01	− 0.51	0.60	− 0.45	− 0.82	0.20	0.32	− 0.82	0.20	0.32
Perforation	0.69	0.17	0.15	0.69	0.27	0.43	0.70	0.43	0.28	0.75	0.42	0.35
Edge	− 0.77	0.48	− 0.33	− 0.86	0.42	0.08	− 0.82	0.17	0.51	− 0.83	0.21	0.46
Loop	− 0.11	0.36	− 0.86	− 0.27	0.60	− 0.73	0.61	0.02	− 0.47	0.54	0.30	− 0.59
Bridge	0.55	0.36	0.11	0.35	0.82	− 0.28	0.76	0.31	− 0.14	0.74	0.14	− 0.30
Branch	− 0.76	0.26	− 0.14	− 0.84	0.47	0.26	− 0.78	0.01	− 0.18	− 0.84	0.47	0.26
Functions												
Built-up area	1.5	− 0.9	0.8	1.8	0.0	1.7	1.6	0.5	0.4	1.6	0.3	0.3
Green area	− 2.8	2.3	− 2.6	− 3.2	6.5	− 3.4	− 1.7	0.0	0.6	− 2.1	0.9	1.0
Arable land	2.8	0.6	2.1	2.8	0.8	1.4	1.6	0.8	0.2	1.6	0.4	− 0.1
Crop mosaic	− 0.1	− 0.8	− 0.2	− 0.1	− 2.5	− 0.1	0.2	− 0.9	− 0.9	− 0.4	− 1.0	− 0.6
Vineyards	0.8	− 0.6	0.3	− 0.1	− 0.7	− 0.3	0.7	− 0.5	− 0.7	0.6	− 1.0	− 1.3
Olive groves	− 2.2	0.4	2.3	− 0.6	− 1.8	− 1.1	− 0.9	− 1.1	− 1.0	0.3	− 1.0	− 1.3
Woodland	1.3	− 0.1	0.4	1.9	1.1	0.3	3.3	2.1	0.2	2.8	2.1	− 0.4
Pastures	1.2	− 0.7	0.8	0.2	− 1.9	− 0.4	− 0.7	− 0.9	− 1.2	0.0	− 1.0	− 1.1
Wetlands	− 2.3	− 0.2	0.7	− 2.6	− 1.7	1.9	− 4.1	− 0.1	2.5	− 4.4	0.4	3.4

Axis 1 identified a structural gradient that separates islets, branches, and edges (negative loadings) from the core, perforation, and bridge patches (positive loadings). This dimension well reflects the processes of landscape fragmentation in the study area. The spatial polarisation in fragmented and pristine land mosaics was more evident at the end of the urbanisation stage (1974). This period was reflective of the highest settlement compactness ever observed in Rome. The corresponding functional gradient associated with Axis 1 separated the most common land-use classes (e.g. built-up settlements, arable land, woodland) in Rome (positive scores) from those more occasionally found in the area (e.g. wetlands, urban gardens/parks, and, partly, olive groves) that received systematically negative scores. In summary, Axis 1 delineates the latent relationship between structure and functions in a metropolitan landscape where the highest degree of patchiness is associated with less common (and more dispersed) land-use classes. In the context of rising human pressure, these classes can be more sensitive to habitat fragmentation.

Reflecting shape and juxtaposition dimensions, Axis 2 highlights the spatial distribution of core patches and the intrinsic relationship with other morphological types (e.g. bridges, loops, islets). This dimension indirectly documents the spatial polarisation in high- and low-fragmentation landscape mosaics typically observed at the end of urbanisation (1974) and reducing gradually in the subsequent observation years (1999 and 2016). From a functional point of view, Axis 2 distinguished anthropogenic land-use classes, such as urban green areas—and classes with medium–low economic value, such as forests—from agricultural classes with medium–high economic potential (e.g. olive groves, pastures, crop mosaics).

Axis 3 reflects landscape connectivity and highlights the role of spatial linkages between patches. For instance, loops (receiving negative loadings in 1949 and 1974) were projected in the opposite factorial quadrant with edges (receiving positive loadings). Reflecting less connected (and possibly more isolated) rural mosaics, land-use classes with a medium–high degree of naturalness (wetlands, olive groves, arable land) received positive scores along Axis 3. In line with these findings, an anthropogenic land-use class such as urban green—well connected with built-up settlements and peri-urban, semi-natural matrices—received positive scores along Axis 3.

Based on these results, MFA provided a multivariate estimation of the intensity of landscape changes characteristic of each developmental stage based on a global correlation coefficient (ranging from 1 to − 1) that indicate, respectively, similarity or dissimilarity in the intrinsic (joint) dynamics of matrices’ rows and columns between observation years. The correlation coefficient between 1949 and 1974 amounted to 0.735, indicating a moderate landscape dynamism during urbanisation. The coefficient declined to 0.622 between 1974 and 1999, indicating a more considerable divergence—and thus accelerated landscape transformations – during suburbanisation. The highest correlation coefficient (0.935) was observed between 1999 and 2016, evidencing less intense dynamics with counter-urbanisation.

### Estimating ‘fast’ and ‘slow’ variables and the rapidity of change in Rome’s landscape

**Figure 2:**
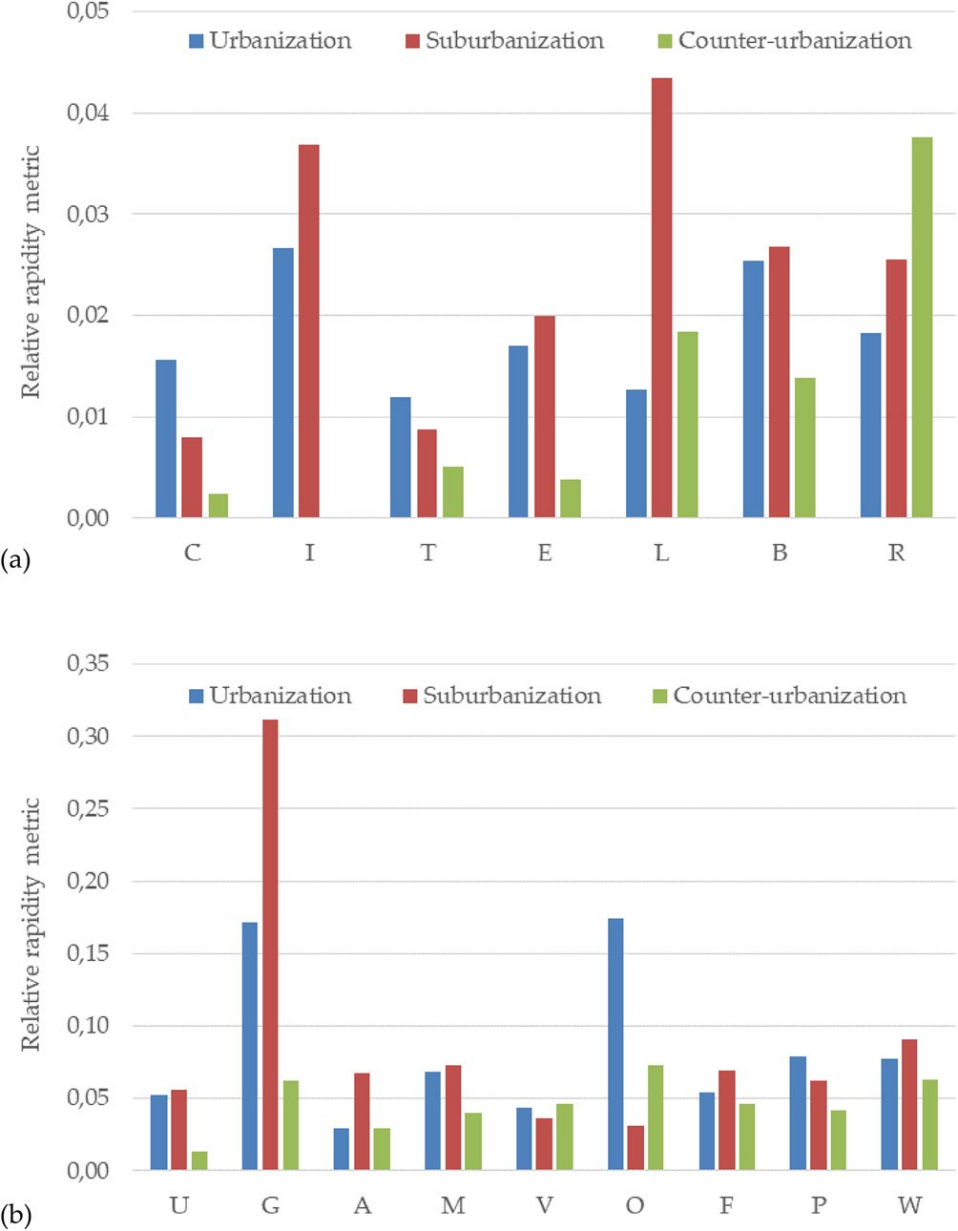
A relative metric of rapidity-of-change estimating ‘fast’ and ‘slow’ attributes of landscape structure (**a**) and functions (**b**), by growth stage in metropolitan Rome. Legend and acronyms: landscape structure (‘C’ = core, ‘I’ = Islet, ‘T’ = Perforation; ‘E’ = Edge; ‘L’ = Loop; ‘B’ = Bridge; ‘R’ = Branch); landscape functions (‘U’ = Built-up area; ‘G’ = Green urban area; ‘A’ = Arable land; ‘M’ = Crop mosaic; ‘V’ = Vineyards; ‘O’ = Olive groves, ‘F’ = Woodland; ‘P’ = Pastures; ‘W’ = Wetlands).

The estimation of rapidity-of-change—seen as an intrinsic, multi-dimensional property of any landscape system—was carried out by introducing a standardised metric derived from computation on the outcomes of the dynamic analysis presented above in “A dynamic analysis of structural and functional indicators of landscape change” section. This metric was calculated separately for each developmental stage for morphological types and land-use classes (Fig. 2). Higher metric values indicate a greater (landscape) dynamism and predisposition to change, possibly reflecting the local background context's evolution.

Considering the structural evolution of Rome’s landscape, rapidity-of-change proved to be systematically lower with counter-urbanisation and higher with urbanisation, particularly with suburbanisation. Core areas experienced limited rates of change instead, declining linearly with time. The structural classes that reflect a more patchy landscape (e.g. islets, loops, bridges, branches) showed the most outstanding dynamism, as they were subject to continuous transformations, particularly evident during suburbanisation. These results document how the structural reorganisation of landscapes facing intense economic pressures because of urban expansion brought more fragmented land mosaics. Considering the three developmental stages—i.e. investigating long-term dynamics that encompass the whole metropolitan cycle from urbanisation to counter-urbanisation—loop, branch and bridge patches totalised the highest value of the metric (respectively 0.010, 0.007 and 0.005) and islets had the lowest value (0.002).

From the functional point of view, a greater dynamism was observed for uncommon land-use (urban green, olive groves, wetlands); contributing the most to landscape changes, they were classified as ‘fast variables’. On the contrary, the most frequent classes in the landscape—or those with a high economic value (e.g. built-up settlements, arable land, vineyards and, partly, crop mosaics, pastures and forests)—experienced a moderate dynamism and were regarded as ‘slow variables’, contributing slightly to landscape change. As for the structural indicators, also in the case of functional indicators, a greater dynamism was associated with suburbanisation (e.g. in the case of urban green) and urbanisation (e.g. in the case of olive groves). Considering the mentioned developmental stages, olive groves, woodlands and wetlands totalised the highest metric values (0.043, 0.041, and 0.035, respectively). On the contrary, crop mosaics assumed the lowest value (0.007).

An overall metric of the rapidity of change was calculated by averaging the individual values associated with structural and functional landscape dynamics indicators. In all cases, the most outstanding dynamism was associated with functional indicators. With urbanisation, the average metric assumed values equal 0.018 (structural indicators) and 0.083 (functional indicators). With suburbanisation, the average metric took on values of 0.024 (structure) and 0.089 (functions). Finally, the metric’s values averaged 0.012 (structure) and 0.046 (functions) during counter-urbanisation. The results of this analysis confirm the greater dynamism associated with suburbanisation as an intrinsic characteristic of the landscape system under investigation.

## Discussion

A diachronic analysis of landscape structure and composition in metropolitan regions provides a detailed assessment of the relationship between settlement morphology and socioeconomic functions^9,15,32,87^. Our study introduces a novel approach based on landscape indicators derived from mathematical morphology, whose outcomes were analysed through a multi-way factor analysis^101^ summarising the latent relationship between form (morphological classes) and functions (land-use). More specifically, the study estimates the net impact of different socioeconomic and territorial configurations on landscape morphology as reflected in three developmental stages: urbanisation, suburbanisation, and counter-urbanisation^63,102,103^. The empirical results of our analysis correctly distinguished these three stages characteristic of Rome’s post-war development trajectory, in line with the outcome of earlier studies^17,73,104^.

Confirming the correspondence between our interpretative model and the (evolving) socioeconomic context in the background^36,78,105^, ‘fast’ and ‘slow’ variables (and the rapidity-of-change characteristic of the landscape system under investigation) were estimated through a dynamic multi-factor analysis using comparable metrics over time^41,106,107^. Our findings document how a multivariate exploratory analysis of landscape indicators may shed further light on latent territorial transformations and diversified socioeconomic contexts^42,61,108^.

Changes in structural and functional indicators allowed a precise characterisation of the three developmental stages mentioned above via specific landscape dynamics. Building compactness and population densification during urbanisation (1949–1974) led to the systematic increase of the mean ‘core’ area of built-up settlements. Settlement dispersion during suburbanisation (1974–1999) led to a systematic reduction in the mean patch size of almost all morphological types. A further—albeit slower—reduction in the mean size of ‘core’ patches (for both urban and non-urban land-use classes) characterised the counter-urbanisation stage (1999–2016). Altogether, these trends reflected the continuous fragmentation of peri-urban mosaics^4,36,77^, and a persistent increase in the fractal dimension of Rome’s landscape, as documented in Salvati^23^.

In these regards, the empirical results of the multivariate analysis translated into a simplified rapidity-of-change metric confirm the role of suburbanisation processes as a catalyst for essential landscape transformations, in line with previous works^109–111^. In the City Life Cycle theory, suburbanisation has often been considered a phase of intense and disordered expansion, driven by diversified (socioeconomic) stimuli and fundamentally unrelated to population increase, the typical growth driver of the ‘compact urbanisation’ stage^20,112,113^. Despite the impact of compact urbanisation, the metropolitan landscape in Rome proved to be much more sensitive to the intrinsic transformations associated with suburbanisation^114^. Socioeconomic impulses typical of ‘compact urbanisation’ exerted an impact mainly on landscape functions, e.g. determining the (radio-centric) expansion of built-up ‘core’ areas and the proportional decline in the size of adjacent (non-urban) patches, arable land^115^. Compact urbanisation dynamics kept the landscape matrix intact in peripheral districts, preserving natural habitats at the local scale and consolidating, at the regional scale, the spatial segregation in urban (high-density) and rural (low-density) areas typical of Mediterranean landscapes^67^.

With suburbanisation, economic impulses and anthropogenic pressures have broadly impacted landscapes' morphological structure, contributing to habitat diversification and fragmentation. This process caused, in turn, a spatial disarticulation of the landscape matrix ^80^, corresponding with the decline of ‘core’ areas and a significant increase in the overall level of patchiness^16,78,116,117^. The multivariate methodology proposed here proved to be effective in interpreting suburbanisation as the primary process at the base of metropolitan landscape transformations in Mediterranean regions^18,84,94^. This result can be generalised to other metropolitan contexts with similar socioeconomic characteristics, such as Barcelona^66^, Athens^69^, Toulouse^59^, Montpellier^73^, Naples^98^, Istanbul^10^, and Adana^1^.

On the contrary, counter-urbanisation—a developmental stage considered, in Mediterranean cities, as more latent and less characterised than urbanisation and suburbanisation^14^—seems to have a minor impact on the evolutionary trajectories of metropolitan landscapes^68^. Subject to constraints of a social and productive nature^63,118,119^, counter-urbanisation in Rome embraced a period of modest growth (early- and mid-2000s) preceding an intense economic crisis (late-2000s) determining the rapid contraction of building activity and the construction market^75^. Felling land and house prices in central areas—combined with a consistent demographic slowdown^64^—have led to more limited land consumption, mainly driven by medium-density urban expansion only along well-defined and broadly accessible development axes^5^.

The approach illustrated in this study may represent an effective tool for monitoring land consumption and informing anti-sprawl policies in metropolitan regions with informal settlements and planning ‘deregulation’ as character traits of their recent development^41,92,106^. In such regions, especially in Mediterranean cities, it was extensively documented how sprawl—recognised as the dominant driver of change—impacted landscape structure and functions for a long time^82^. In this context, the operational framework proposed here may support planning strategies to contain sprawl by preserving sustainable (e.g. compact and land-saving) urban forms^84^. At the same time, the results of our study confirm the importance of developmental policies consolidating economically dynamic and socially cohesive settlement models in peripheral districts^120^. From a structural perspective, these policies should also reconnect semi-dense and sparse settlements with the high-quality agro-forest matrix typical of Mediterranean landscapes into a balanced mosaic mixing urban and rural functions. Based on the proposed logical approach, future studies can implement a comparative analysis of long-term landscape dynamics along an urban hierarchy (e.g., large cities to medium-sized towns) to unveil the effectiveness of spatial planning and anti-sprawl policies at different spatial scales and in largely variable socioeconomic contexts^25^.

From a technical perspective, the exploratory (multivariate) techniques adopted in this study (mixing a mathematical morphology approach with a dynamic factor analysis) provide a simplified, flexible and adaptive interpretation of multi-dimensional landscape changes^121^. However, the rising heterogeneity of socio-demographic processes over space, and their impact on landscape structure and functions in metropolitan regions of advanced economies^122^, justify the implementation of more structured exploratory methodologies and soft modelling addressing the incipient complexity and fractality of urban systems^93^. Thanks to the increasing availability of geo-spatial databases^123^, future studies reconnecting traditional exploratory techniques (multivariate statistics, classification and regression trees) with informatics/cybernetic approaches (advanced data mining procedures, neural networks, and machine learning), are recommendable in the field of automatic recognition of a complex (socioeconomic and environmental) pattern of change, as a relevant contribution to landscape science.

## Conclusions

The ‘complex system’ vision adopted in this study allows a thorough investigation of the (evolving) relationship between landscape form and functions, contributing to design (or re-design) planning strategies able to face socioeconomic transformations as the main engine of landscape complexity and fragmentation processes. Linking economic change and social dynamics to landscape modifications in a diachronic perspective, i.e. interpreting the present (landscape) structure as a function of past development, provides appropriate knowledge to any strategy promoting resilience and sustainability in metropolitan areas.

## Data Availability

The datasets analysed during the current study are available from the corresponding author upon reasonable request.
